# Rivaroxaban equals warfarin treatment in atrial fibrillation patients at high risk of stroke

**Published:** 2010-12

Rivaroxaban has met its primary objective in the ROCKET trial of non–inferiority to warfarin in the treatment of atrial fibrillation (AF) patients at high risk of stroke. In fact the study included a majority of patients who had already experienced either a stroke or transient ischaemic event, therefore needing warfarin for secondary prevention.

For the primary–efficacy endpoint, rivaroxaban was superior to warfarin, delivering a 21% relative risk reduction in stroke and non–central nervous system (CNS) systemic embolism in the prespecified on–treatment population (1.70 vs 2.15%, p = 0.015).

Could these results of rivaroxaban in the ROCKET trial have been foreseen by Prof John Camm, University of London, in his commentary a year ago on the RELY study?^1^ He pointed out then, ‘much more information will be needed before regulators can decide on the approvability of the drug [in this case, he was referring to dabigatran] for the management of patients with atrial fibrillation with thrombo–embolic risk (CHADS score greater than 2%)’.

We now know that the FDA has approved the higher dose of dabigatran (150 mg twice daily) to prevent stroke in patients with atrial fibrillation, and a 75–mg dose for patients with severe renal impairment (15–30 ml/mm). The FDA made no additional stipulations for dabigatran usage in AF except to say, ‘for stroke prevention’; while the Canadian approval says, ‘for AF patients in Canada in whom anti–coagulation is appropriate’.

The randomised, double–blind ROCKET study could make an argument for the usage of rivaroxaban in patients with AF and a high CHADS score. In ROCKET, the majority of patients were in the 3–5% CHADS score risk assessment, with a mean of 3.48% (Table 1). The CHADS score in the RELY study was 2.1%. The primary efficacy outcome for the ROCKET study is shown in Fig. 1 and Table 2.

**Fig. 1:**
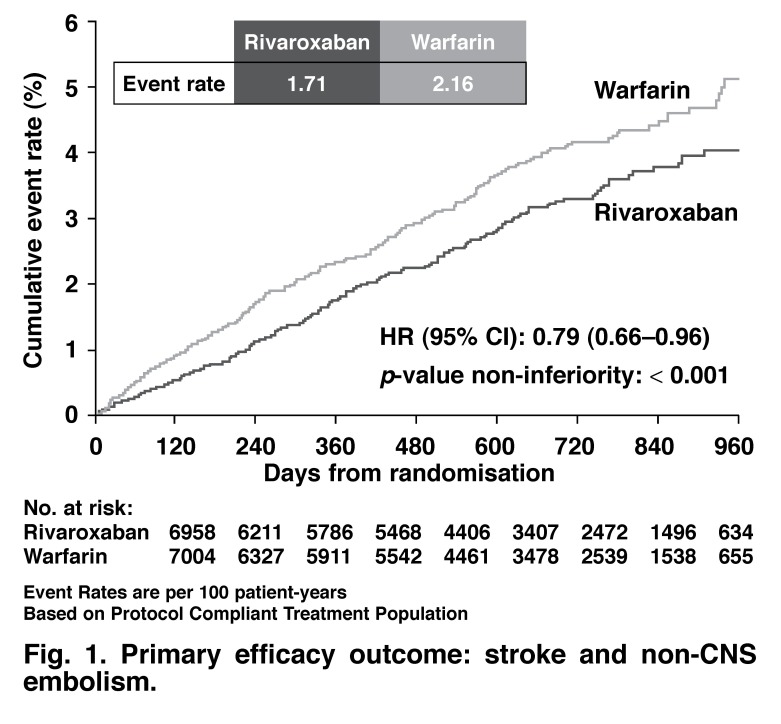
Primary efficacy outcome: stroke and non-CNS embolism.

**Table 1:** 1BASELINE DEMOGRAPHICS

	*Rivaroxaban (n = 7 081)*	*Warfarin (n = 7 090)*
CHADS_2_ score (mean)	3.48	3.46
2 (%)	13	13
3 (%)	43	44
4 (%)	29	28
5 (%)	13	12
6 (%)	2	2
Prior VKA use (%)	62	63
Congestive heart failure	63	62
Hypertension (%)	90	91
Diabetes mellitus (%)	40	39
Prior stroke/TIA/embolism (%)	55	55
Prior myocardial infarction (%)	17	18

Based on intention-to-treat population

**Table 2:** PRIMARY EFFICACY OUTCOME: STROKE AND NON-CNS EMBOLISM

*Rivaroxaban event rate*	*Warfarin event rate*	*HR (95% CI)*	*p-value*
1.70	2.15	0.79 (0.65–0.95)	0.015
2.12	2.42	0.88 (0.74–1.03)	0.117

Event rates are per 100 patient years

Based on safety on treatment or intention-to-treat through site-notification populations

The event rate for stroke and non–CNS embolism was 1.7 per 100 patient years for rivaroxaban and 2.15 for warfarin, based on the on–treatment population. The intention–to–treat (ITT) event rates were higher; 2.12 with rivaroxaban and 2.42 for warfarin.

In ROCKET, the time in therapeutic INR range was 57.8%. There were similar rates of bleeding and adverse events in the rivaroxaban and warfarin arms, but less intra–cranial haemorrhage and fatal bleeding with rivaroxaban. Prof Kenneth W Maheffey of Duke University concluded that rivaroxaban has now been shown to be a proven alternative to warfarin for stroke prevention in moderate– or high–risk patients with non–valvular AF.

South Africa participated in the ROCKET study and entered 247 patients into the study.

The discussant of this study at the American Heart Association, Dr Elaine M Hylek, Boston, USA pointed out that ROCKET had recruited the oldest and highest–risk AF patient cohort, with 55% having had a stroke and therefore requiring warfarin for secondary prevention, 91% with hypertension, 62% with chronic heart failure (which would have contributed to INR variability), and 39% with diabetes. ‘These were patients at the highest risk for intra–cranial haemorrhage due to age, high blood pressure and prior stroke’, she pointed out.

In this high–risk population, less than 50% achieved the time–in–therapeuticrange (TTR) threshold of >58–60% needed to realise the benefits of warfarin. She pointed out that per–protocol or ‘on–treatment analysis’ is important to confirm non–inferiority of a primary intention–totreat analysis. Then, after non–inferiority is evident, superiority may be assessed, preferably defined at the outset and with an intention–to–treat analysis.^2^ Dr Hylek’s conclusions are summarised:

- Based on both the ITT and per protocol (PP) analysis, rivaroxaban is non– inferior to warfarin (albeit with TTR = 57%) for the prevention of stroke in AF patients.
- The difference between the ITT and per protocol analysis may be accounted for by poor adherence. This raises concerns about the relevance of the PP analysis to real–world practice, particularly for a drug with a half–life of 5–13 hours vs 20–60 hours for warfarin.
- The ITT result showing no significant superiority is more likely to reflect the actual difference in effectiveness between these treatments
- Importantly, there were fewer intracranial bleeds on rivaroxaban and fewer deaths from bleeding. However, there were more haemorrhages requiring transfusions, and drops in haematocrit, rendering the overall safety profile less clear.

## Additional comments from an interview with Dr Jonathan Halperin, Mount Sinai School of Medicine, New York, USA, who attended the hot line session

It is very hard to draw comparisons between the two trials, ROCKET-AF and RELY, as ROCKET enrolment specifically stipulated the inclusion of high-risk patients – hence the difference in mean CHADS_2_ risk scores between ROCKET (~ 3.5) and RELY (2.1). In addition, methodological differences confound comparison: ROCKET was a doubleblind study while RELY was an open study with blinded endpoint assessment (probe design).

There was great anticipation about this study, particularly as a press release from Europe indicated that the study had reached its primary objective to demonstrate the non-inferiority of rivaroxaban compared to warfarin. The lead organisation, the Duke Clinical Trials Institute, has done a remarkable job in analysing and organising these data just a few weeks after closure of the trial database. This means, however, that the results presented at this congress are top-line findings, and we certainly anticipate and perhaps even hunger for more detail.

One of the surprising findings of the trial is that warfarin did not perform as well in this double-blind trial (achieving a TTR below 60%) as in the North American SPORTIF-V trial of another anticoagulant, later abandoned due to liver toxicity, in which warfarin’s TTR was 68%. The reasons for the poorer warfarin control in ROCKET-AF could include the high-risk patient profile or the inclusion of geographically diverse centres, some of which may have more experience with vitamin K antagonists other than warfarin.

The good news is the development of another effective anticoagulant, one that inhibits activated factor X, demonstrating non-inferior efficacy compared to warfarin, with lower rates of intra-cerebral haemorrhage. Concern remains about whether the results may have been different if the quality of warfarin control had been better, and about higher bleeding rates outside the central nervous system with rivaroxaban, leading to comparable rates of major bleeding with both treatments overall.
